# Understanding the Role of Human Papillomavirus in Head and Neck Cancer of Unknown Primary: A Systematic Review

**DOI:** 10.7759/cureus.39643

**Published:** 2023-05-29

**Authors:** Tomas Escobar Gil, Maria A Henao Rincón, Juanita Laverde, Alejandro Echavarria Cross, Carlos S Duque

**Affiliations:** 1 Internal Medicine, University of New Mexico School of Medicine, Albuquerque, USA; 2 Otolaryngology, Universidad de Cartagena, Cartagena de Indias, COL; 3 Internal Medicine, Universidad Ciencias de la Salud (CES), Medellín, COL; 4 Otolaryngology, Hospital Pablo Tobón Uribe, Medellín, COL

**Keywords:** general otolaryngology, head and neck tumors, unknown primary site, oncology, otolaryngology-head and neck surgery, human papillomavirus, cancer of unknown primary, cancer, head and neck

## Abstract

This systematic review aims to provide a comprehensive understanding of the role of human papillomavirus (HPV) in head and neck cancer of unknown primary (HNCUP). HNCUP is a rare type of cancer with an unknown primary site, which makes it difficult to diagnose and treat. The review includes articles published between 2013 and 2023 that investigated the prevalence of HPV in HNCUP, its association with clinical outcomes, and its potential implications for diagnosis and treatment. The search was conducted in 11 electronic databases, and the gray literature: Cochrane, Cumed, IBECS, JAMA Network, LILACS, MEDLINE Ovid, MEDLINE-EBSCO, PubMed, Scopus, SciELO, and Taylor & Francis Online; a total of 23 studies met the inclusion criteria. The review found that HPV is present in a significant proportion of HNCUP cases, ranging from 15.5% to 100%. HNCUP incidence is increasing, and the presence of HPV was associated with improved clinical outcomes in some studies, such as overall survival and disease-free survival; but was found to have no association with outcomes in others. This may have implications for diagnostic and treatment strategies. The findings of this review suggest that further research is needed to better understand the role of HPV in HNCUP and to develop targeted therapies for this disease.

## Introduction and background

**Figure 1:**
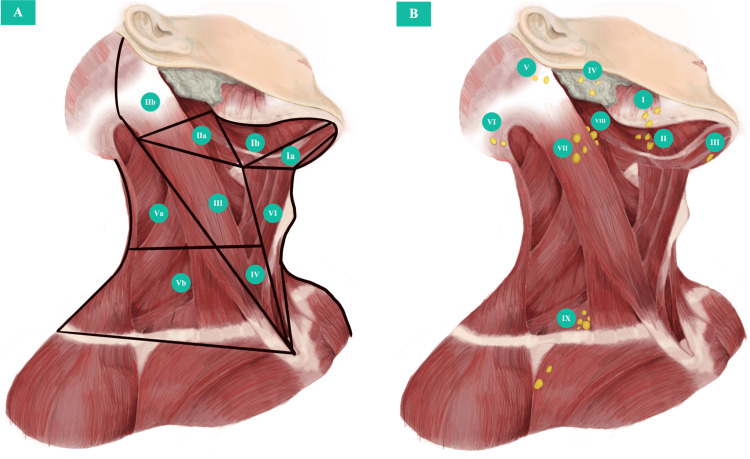
Lymphatic system of the head and neck This illustration is the authors' own creation. The first levels of lymphatic drainage according to the head and neck site (Figure 1A), and the superficial cervical lymph node chains (Figure 1B) are depicted. Figure 1A. Zones Ia, Ib: oral cavity, lip, submaxillary gland. Zones IIa, III, IV: oral cavity, oropharynx, larynx, hypopharynx, thyroid. Zone IIb: nasopharynx, parotid gland, oropharynx. Zones Va, Vb: nasopharynx, thyroid. Zone VI: thyroid, larynx, hypopharynx, cervical esophagus. Figure 1B. I: supramandibular. II: submandibular. III: submental. IV: parotid. V: mastoid. VI: occipital. VII: superficial cervical. VIII: superior deep cervical. IX: inferior deep cervical.

Head and neck cancers of unknown primary (HNCUP) pose a diagnostic challenge for clinicians. They are characterized by the presence of metastatic disease in the neck lymph nodes without any evidence of a primary tumor in the upper aerodigestive tract, despite thorough investigation [1]. These tumors account for 1% to 4% of all head and neck cancers, and their incidence is rising. Unfortunately, their clinical prognosis is generally poor due to high recurrence rates and limited treatment options [2]. Therapeutic approaches include neck dissection alone, neck dissection followed by radiation with or without concurrent chemotherapy, or primary chemoradiation according to initial nodal disease. Whether the potential mucosal primary site and/or the contralateral neck should be electively treated is controversial [2]. While the location of the metastatic lymph node does not always indicate a specific origin, it can sometimes provide clues about the potential primary site of the tumor [1,2]. This is depicted in Figures 1A, 1B.

Some studies have suggested a potential role for human papillomavirus (HPV) in the development of head and neck cancer of unknown primary (HNCUP); however, the exact mechanisms and prevalence of HPV in these tumors remain unclear [3]. There is an increasing understanding of the implications of HPV on the diagnosis, treatment, and patient outcomes of certain head and neck cancer types, such as squamous cell oropharyngeal carcinoma (SCOPC). The most common site of a small primary tumor initially thought to represent a HNCUP is the tonsil or base of the tongue and an increasing percentage are associated with human papillomavirus [4]. These advancements have led to changes in current guidelines [4]. Nonetheless, there are still gaps in knowledge, particularly in Latin America, and further research on this topic is necessary [5].

The objective of this present study is to evaluate the existing literature regarding the role of HPV in HNCUP. Specifically, the study aims to assess the prevalence of HPV, its association with clinical outcomes, and its impact on patient prognosis. Through a systematic review of published and gray literature, studies meeting predetermined inclusion and exclusion criteria were selected. Relevant information was extracted, and subsequent analyses were performed to address the study objective. This systematic review is important as it provides a comprehensive overview of the current evidence on the role of HPV in HNCUP. The findings will have implications for therapeutic management in patients with this challenging condition and will guide future research by enhancing our understanding of the tumors' pathogenesis and identifying new avenues for diagnosis and treatment.

## Review

Materials and methods

To design this systematic review, a thorough literature search using the Critical Appraisal Skills Programme (CASP) guidelines were used [6], and to construct the flowchart of the methodology and research process, the Preferred Reporting Items for Systematic Reviews and Meta-Analyses (PRISMA) statement was used as a model [7].

Information was gathered from 11 databases and the gray literature: Cochrane, Cumed, IBECS, JAMA Network, LILACS, MEDLINE Ovid, MEDLINE-EBSCO, PubMed, Scopus, SciELO, and Taylor & Francis Online. *Descriptores en Ciencias de la Salud* (DeCS) and Medical Subject Heading (MeSH) terms included the following: "head and neck", "unknown primary tumor", "occult primary", "HPV", "human papillomavirus", "virus del papiloma humano" "cabeza y cuello", "cáncer de origen desconocido", "primario oculto", and "VPH". Boolean operators "AND", "Y", "OR", and "O", were used as linking words. We only considered articles published between 2013 and 2023, and filters were applied to retrieve results in English or Spanish only. The articles that the databases yielded were then screened by title and abstract.

Only studies with original data were considered when choosing the study types. Articles that assessed the link between HNCUP (or SCCUP) and HPV, their prevalence, correlation with clinical outcomes, and influence on patient prognosis were chosen. The analysis was limited to research in humans and only included case-control studies, randomized clinical trials, cross-sectional studies, retrospective or prospective cohort studies, and cohort studies.

The exclusion criteria included: joint committees; guidelines; classifications; algorithms; studies about *Mycoplasma hominis* and HNCUP, Epstein-Barr and HNCUP, or microorganisms other than HPV; articles about HPV-negative carcinomas of the head and neck only, or oropharyngeal cancer (OPC) only; works on public policy; studies of HPV-related cancers of known origin; comments and letters to the editor; and texts on HPV-genotyping. Furthermore, only articles that satisfied all inclusion criteria had their full texts examined. Complete books, mini-reviews, meta-analyses, opinion pieces, systematic review articles, and letters to the editor were not considered.

Results

**Figure 2:**
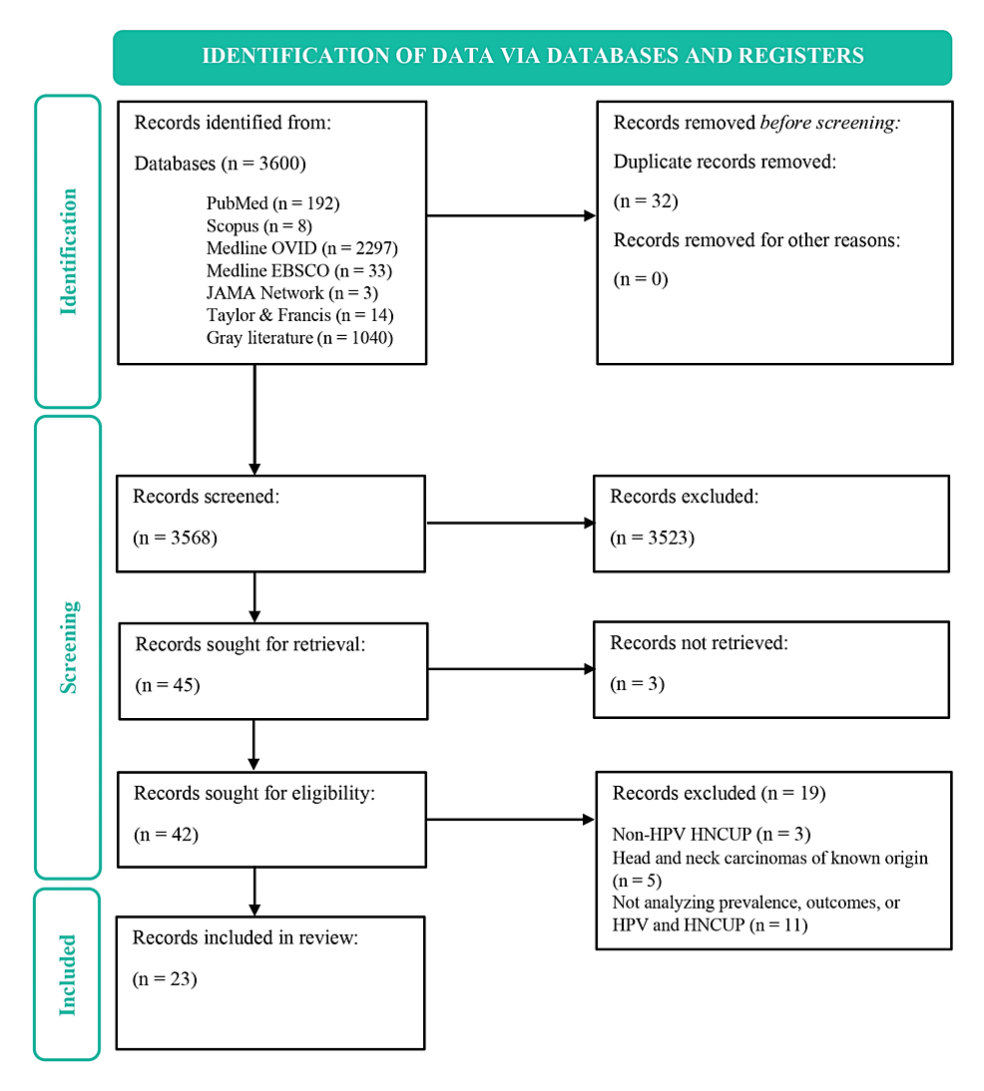
Flowchart for article selection HPV - human papillomavirus, HNCUP - head and neck cancer of unknown primary

Through database searching, a total of 3600 records were found. Forty-two were chosen for full-text review after deleting duplicates, records older than 10 years, and articles written in languages other than English and Spanish, in addition to examining titles and abstracts. Nineteen of the latter were eliminated using exclusion criteria. Twenty-three papers were consequently included in the systematic review. This process can be found in greater detail in Table 1 and Figure 2.

**Table 1:** Search results in databases MeSH - Medical Subject Heading; DeCS - Descriptores en Ciencias de la Salud; HPV - human papillomavirus; VPH - virus del papiloma humano

Database	MeSH and DeC terms used	Number of articles
PubMed	[("Head and neck")] AND [("Unknown primary tumor") OR ("Occult primary")] AND [("HPV") OR ("Human papillomavirus")]	192
Cochrane	[("Head and neck")] AND [("Unknown primary tumor") OR ("Occult primary")] AND [("HPV") OR ("Human papillomavirus")]	0
Lilacs	[("Cabeza y cuello")] Y [("Cáncer de origen desconocido") O ("Primario oculto")] Y [("VPH") O ("Virus del papiloma humano")]	0
IBECS	[("Cabeza y cuello")] Y [("Cáncer de origen desconocido") O ("Primario oculto")] Y [("VPH") O ("Virus del papiloma humano")]	0
Medline Ovid	[("Head and neck")] AND [("Unknown primary tumor") OR ("Occult primary")] AND [("HPV") OR ("Human papillomavirus")]	2297
Cumed	[("Cabeza y cuello")] Y [("Cáncer de origen desconocido") O ("Primario oculto")] Y [("VPH") O ("Virus del papiloma humano")]	0
JAMA Network	[("Head and neck")] AND [("Unknown primary tumor") OR ("Occult primary")] AND [("HPV") OR ("Human papillomavirus")]	3
Scopus	[("Head and neck")] AND [("Unknown primary tumor") OR ("Occult primary")] AND [("HPV") OR ("Human papillomavirus")]	121
Scielo	[("Cabeza y cuello")] Y [("Cáncer de origen desconocido") O ("Primario oculto")] Y [("VPH") O ("Virus del papiloma humano")]	0
Medline EBSCO	[("Head and neck")] AND [("Unknown primary tumor") OR ("Occult primary")] AND [("HPV") OR ("Human papillomavirus")]	33
Taylor & Francis Online	[("Head and neck")] AND [("Unknown primary tumor") OR ("Occult primary")] AND [("HPV") OR ("Human papillomavirus")]	14
Gray Literature (Google Scholar)	[("Head and neck")] AND [("Unknown primary tumor") OR ("Occult primary")] AND [("HPV") OR ("Human papillomavirus")]	1040

Titles and abstracts were independently evaluated by two authors to determine eligibility for full-text review. Discussions were used to settle disagreements. To incorporate research that had not been found in the electronic or gray literature review, the references of pertinent reviews and those of chosen articles were manually examined. The studies [8-30], their authors, the nation in which they were published, the number of patients, mean age, gender, and HPV status were examined and compiled into Table 2.

**Table 2:** Relevant studies exploring the role of HPV in HNCUP DFS - disease-free survival; DNA - deoxyribonucleic acid; ECE - extracapsular extension; F - female; FNAC - fine-needle aspiration cytology; FNA - fine-needle aspiration; HNCUP - head and neck cancer of unknown primary; HNSCC - head and neck squamous cell carcinoma; HPV - human papillomavirus; HPV+ - positive human papillomavirus; HPV- - negative human papillomavirus; IHC - immunohistochemistry; M - male; mRNA - messenger ribonucleic acid; N/A - not available; ND - node dissection; OPC - oropharyngeal carcinoma; OS - overall survival; PCR - polymerase chain reaction; PI3K - phosphoinositide 3-kinase; RT - radiotherapy; TP53 - tumor protein P53; USA - United States of America; TNM - tumor, node, metastasis classification; TSG - tumor suppressor gene; X̄ - mean

Authors	Country	Number of patients	Gender	Mean age	HPV+ (DNA or P16)	HPV-	Variable studied	Results of the study
Axelsson et al. [8]	Sweden	n = 260	F: 27%, M: 73%	X̄ = 34	81%	18%	Compare the two main treatments for HNCUP: neck dissection and (chemo) radiation vs primary (chemo) radiation.	Survival for curatively treated patients with HPV+ and HPV-HNCUP did not differ significantly.
Axelsson et al. [9]	Sweden	n = 68	F: 45%, M: 55%	X̄ = 59.4	69%	18% (p16 analysis not possible in 13%)	Evaluate the prognostic importance of different factors, including HPV status, treatment, and overall survival.	Curatively treated HNCUP had good survival. Independent prognostic factors for survival were age over 70 years, HPV status, and N3 stage.
Balk et al. [10]	Germany	n = 80	F: 4.2%, M: 95.8%	X̄ = 60.6	73.8%	26.2%	Assesses the impact of surgical and non-surgical treatment modalities and tumor biology on the oncological outcome.	The authors were not able to demonstrate any advantage in the multiple analysis either for HPV+ or HPV-HNCUPs.
Bersani et al. [11]	Sweden	n = 325	F: 23%, M: 77%	X̄ = 60	85.8%	14.2%	Evaluate predictive markers for response to treatment, and correlations and differences in mutated oncogenes and TSG between HPV+ and HPV- HNCUP.	Mutations/tumors were fewer in HPV+ HNCUP, compared to HPV- tumors. Differences in mutation frequency of TP53 and PIK3CA were found between HPV+ HNCUP and HPV-.
Bussu Solo et al. [12]	Italy	n = 22	F: 14%, M: 86%	X̄ = 62	45.4%	54.6%	Evaluate the prevalence of several viruses in neck metastases from HNCUP.	No significant correlations between virus detection and clinicopathologic parameters or prognosis.
Channir et al. [13]	Denmark	n = 93, 8 with HNCUP	F: 22%, M: 78%	X̄ = 60.7	N/A	N/A	Assess PCR-based HPV DNA testing on FNA smears in a clinical setting.	HPV DNA testing on FNA smears can be performed within a reasonable timeframe and can guide the detection of an HPV+ OPC.
Cheraghlou et al. [14]	USA	n = 972	F: 16%, M: 84%	X̄ = not specified	76.3%	24.7%	Evaluate the treatment-related outcomes for HPV+ and HPV-HNCUP.	Tumor HPV status has a significant prognostic value for HNCUP and should be considered in future studies of treatment de-intensification in this group.
Cummings et al. [15]	USA	n = 964	F: 21.9%, M: 78.1%	X̄ = 57	31.7%	9.9% (58.2% was unknown/not reported)	Describe the changing incidence of HNCUP and assess diagnostic and treatment strategies.	The incidence of unknown primary head and neck carcinoma is increasing, and current cases have a high proportion of HPV positivity.
Davis et al. [16]	USA	n = 44	F: 4.5%, M: 95.5%	X̄ = 57	95.5%	4.5%	Determine if identification of the primary tumor is associated with improved oncologic outcomes and/or tumor characteristics including HPV status.	HPV positivity is associated with the discovery of the primary tumor. Discovery of the primary lesion is associated with improved overall prognosis.
Demiroz et al. [17]	USA	n = 41	F: 10%, M: 90%	X̄ = 53	32%	45% (23% not available)	Characterize HNCUP and retrospectively compare outcomes for patients treated with ND+RT versus definitive RT.	Neck dissection and post-op RT resulted in a similar outcome as definitive RT. The prognostic implications of HPV+ nodes in HNCUP are similar to those in OPC.
Dixon et al. [18]	Canada	n = 73	F: 12%, M: 88%	X̄ = 61	63%	37%	Evaluate the prognostic significance of HPV in the context of HNCUP.	Among patients with HNCUP, p16-positive status is an independent predictor of DFS but not OS.
Jensen et al. [19]	Denmark	n = 60	F: 24.4%, M: 76.6%	N/A	55%	45%	Examine the prevalence of HPV in patients with HNCUP.	A fairly large percentage of HNCUP cases are HPV-related and HPV testing is recommended as part of the diagnostic workup.
Keller et al. [20]	USA	n = 39	F: 17%, M: 83%	N/A	74%	26%	Evaluate HPV status and study biomarkers potentially prognostic in HNCUP.	The majority of HNCUP patients were p16+, indicative of HPV association.
McDowell et al. [21]	Australia	n = 143	F: 10%, M: 90%	X̄ = 76	46%	54%	Evaluate for p16 expression, detect 18 high-risk HPV subtypes, and correlate results with clinicopathological features and outcomes.	HPV testing may provide additional information for determining a putative primary site.
Motz et al. [22]	USA	n = 84	F: 11.9%, M: 88.1%	X̄ = 57.3	82%	18%	Determine the frequency of HNCUP over time and evaluate the proportion of HPV-positive HNCUP.	The frequency of HNCUP has increased significantly. As expected, patients with HPV-positive HNCUP tend to be male and younger.
Rollo et al. [23]	Italy	n = 63	F: 32%, M: 68%	X̄ = 61	66%	34%	HPV presence was evaluated in FNA collected from patients with OPC or HNCUP.	In patients with initial HNCUP, HPV‐positivity on the FNA may guide the diagnostic workup and therapeutic management.
Ross et al. [24]	USA	n = 96	F: 7%, M: 93%	X̄ = 56.1	100%	0%	Compare outcomes between T0N1-3M0 HPV+ HNCUP and T1-2N1-3M0 HPV+ OPC.	Patients with T0N1M0 HPV+ HNCUP have similar survival outcomes to matched patients with T12N1M0 HPV+ OPC.
Schroeder et al. [25]	Germany, Italy, and Spain	n = 180	F: 12%, M: 88%	X̄ = 61	15.5%	84.5%	Determine if the detection of markers for HPV transformation in HNCUP could be of value for choice and extent of treatment.	HPV-driven HNCUP had significantly better survival rates. HPV RNA status should be included in HNCUP diagnosis and therapy.
Schroeder et al. [26]	United Kingdom	n = 196 patients with HNCUP	Predominantly male	N/A	68%	32%	To compare risk factors and survival in people with oropharyngeal cancer OPC and HNCUP.	HPV-driven HNCUP is likely to be HPV-driven OPC.
Shan et al. [27]	USA	n = 20	F: 0%, M: 100%	X̄ = 57	100%	0%	Examine the potential benefit of reevaluation of original slides and p16 IHC of tonsillectomy specimens for primary tumor identification in cases of HPV+ HNCUP.	Re-review of original slides by an expert head and neck pathologist, but not p16 staining or deeper H&E sections, was able to identify additional tumors.
Sivars et al. [28]	Sweden	n = 50	F: 26%, M: 74%	X̄ = 65	80%	20%	Investigate whether HPV status and p53-expression correlated to clinical outcomes in patients with HNCUP.	Both HPV status and p53 expression are valuable prognostic factors in patients with HNCUP and should be further explored for clinical use.
Takes et al. [29]	The Netherlands	n = 47	F: 30%, M: 70%	X̄ = 58	53%	47%	Assess HPV status on FNAC and validate it using histological material of the same patients.	Testing on HPV in FNAC of cervical lymph node metastases of SCC is validated.
Wagner et al. [30]	Germany	n = 103	F: 24%, M: 76%	X̄ = 62.9	31%	69%	Assess a consecutive cohort of HNCUP for HPV DNA, mRNA, p16 expression, and risk factors to identify prognostic classification markers.	Despite obvious differences, HNCUP shares similarities in risk profile with OPC.

The articles selected were mostly written in Europe and the United States: four articles in Sweden [8,9,11,28], two in Germany [10,30], two in Italy [12,23], two in Denmark [13,19], eight in the United States [14-17,20,22,24,27], one in Canada [18], one in Australia [21], one in the United Kingdom [26], one in the Netherlands [29], and one in various European countries [25].

Each study had an average of 301.57 participants, ranging from 20 in the study with the fewest patients [27] to 972 in the one with the most [14]. In every study, there was a male predominance, including one where 100% of participants were men [27]. The study with the highest female representation was 45% [9]. The mean age of participants was 54.5, with the majority of patients falling in the range of the sixth and eighth decades of life. Four studies did not report the age of patients, or it was not specified [14,19,20,26].

The methods used to assess HPV status in the studies were p16 and DNA, and all studies but one [27] reported the percentage of patients with HPV positivity. Studies focused primarily on the outcomes of these patients, their epidemiological factors, and their prognosis.

Discussion

HNCUP Incidence

Two studies evaluated this variable and suggested that the incidence of HNCUP is increasing [15], with one explanation being the increasing detection of HPV-positive disease. This is consistent with the findings in our systematic review, where the majority of patients with HNCUP had positive HPV results. Another author [22], who came across similar findings, added that the profile of patients with HNCUP is commonly a young male patient, which is another feature consistent with our review, as in every study, there was a middle-age male predominance.

HPV is implicated in the pathogenesis of SCOPC due to mechanisms that are not fully understood, and a vast proportion of HNCUP end up being SCOPC when more information becomes available, which makes the assessment of HPV in patients with HNCUP even more imperative [4,19,20,23,26,30].

Assessing the HPV Status of Patients with HNCUP

Screening for association with HPV has been part of the HNCUP workup procedure since the 2017 8th edition of the American Joint Committee on Cancer (AJCC)'s Tumor Node Metastasis (TNM) classification [4,31]. It classifies unknown primaries associated with HPV as being of oropharyngeal origin. This approach helps guide treatment and, notably, to select target mucosa volumes in radiation therapy [31].

Most of the studies in this review demonstrated positivity for HPV in a range between 15.5% [25] and 100% [24,27], with a mean of 65.42%; this variable was not measured in one of the articles [27]. Common methods to assess HPV status are direct (like in situ hybridization or polymerase chain reaction (PCR) methods) or indirect (like p16), as the overexpression of this tumor suppressor protein has been consistently documented (>95%) in some HNCUP, and it has been suggested as a surrogate marker for HPV-associated tumors [32]. In this systematic review, the studies heterogeneously employed both techniques.

Two of the studies suggested additional benefits of using p16, such as acting as a predictor of added disease-free survival (DFS) independent of nodal status and treatment [18,20]. However, another study found that it was not prognostic for overall, cancer-specific, or progression-free survival [21]. Moreover, p16 positivity is also correlated to SCOPC, and all the above studies suggested it yields important information when locating the primary tumor [18,21]. Another study evaluated the time spent and availability of HPV DNA testing and concluded that it can be performed within a reasonable timeframe and can guide the detection of an HPV-related SCOPC in the context of HNCUP [13]. Additionally, one of the authors concluded that the assessment of 18 high-risk HPV types provides additional information for determining a putative primary site and correlates the HNCUP with a possible SCOPC [21]. Lastly, one of the studies suggested that patients with HNCUP may benefit from ribonucleic acid (RNA) HPV testing as HPV status may impact survival and influence diagnosis and therapeutic decision-making [25].

The use of direct or indirect techniques should be determined on a case-by-case basis and considering the availability of each, as there does not seem to be a significant difference in their overall performance [12]. Many countries, especially in Latin America, have scarce resources, so clinicians must consider this, and if only p16 is available, it should be used [5]. What is certain in this matter is that the workup of HNCUP requires HPV testing to help locate the primary tumor [19].

Other Diagnostic Considerations

Other variables explored by the studies in this review included the reassessment of pathology slides by specialized head and neck pathologists when there were doubts about the diagnosis of HNCUP [27]. The authors determined that a re-review of original slides by an expert head and neck pathologist allowed the identification of additional tumors. This may shed light on cases where the diagnosis is uncertain and more specialized pathologists are available.

Another work explored the utility of biopsies performed in cervical lymph node metastases, using patients as their own controls by comparing the results to the histological material of the same patients [29]. Testing on HPV in fine-needle aspiration cytology (FNAC) of cervical lymph node metastases of squamous cell carcinoma (SCC) is validated according to this study. This could further impact metastatic cases where biopsies could otherwise not be performed due to complexity or anatomic location.

Advances are being made at the biomolecular level with the analysis of mutations of HPV-related tumors compared to HPV-negative tumors [11,28]. In a study done in Sweden [11], mutations per tumor (MPT) were analyzed, and it was found that there were fewer MPT in HPV-positive HNCUP compared to HPV-nonrelated tumors. Differences in mutation frequency of tumor protein 53 (TP53) and phosphoinositide 3-kinase (PIK3CA) were found between HPV-related HNCUP and HPV-nonrelated tumors, along with other mutations in both profiles. The findings of this study are important, as mutation assessment could impact patient prognosis [11]. The work of another author suggested that TP53 expression is a valuable prognostic factor in patients with HNCUP and should be further explored for clinical use [28]. More biomolecular research is needed to further explore the mutations of HPV-related tumors and their overall impact on the disease and its outcomes.

Prognosis and Outcomes

Prognostic implications of HPV in the context of HNCUP are, perhaps, the point that brings up the most controversy within studies. Some authors did not find any difference in the prognosis of patients regarding HPV status [8,10,17,19], while some did, and even more so, suggested the possibility of de-intensification of treatment for this patient group [9,14,16,25]. Axelsson et al., specifically, reported that curatively treated HNCUP has a good survival, with an overall five-year survival rate of 82%, and found that p16 positivity is associated with significantly longer survival and a lower risk of tumor recurrence [8]. Although it is not entirely clear why the improvement in the prognosis, some reports mention that it is probably due to a better discovery of the primary tumor in association with HPV positivity [16], in addition to the fact that the immunology of tumors caused by HPV is more susceptible to treatment. Another author found mixed results [18] and determined that among patients with HNCUP, p16-positive status is an independent predictor of disease-free survival (DFS) but not overall survival (OS).

In addition to HPV, some studies have identified additional prognostic factors for survival, such as age over 70 and N3 stage, which are considered independent factors [16]. Other studies highlight that the N stage is a significant prognostic factor for overall survival in patients with HNCUP treated with curative intent [9]. In another study, the main poor prognostic factor was the development of distant metastases [10].

Further studies or meta-analyses that include larger samples should be performed to delve further into this. Some studies also highlighted the lack of difference in the efficacy of treatment modalities (like neck dissection and definitive radiotherapy) in patients with HNCUP [8,17].

TNM Status

A lower age has been consistently linked to improved survival outcomes. Reports have varied in terms of the importance attributed to the N stage, with some authors finding statistically worse survival for N3, while others have identified N2b, N2c, and N3 as contributing factors. Additionally, decreasing survival trends have been observed for N1, N2, and N3 stages in certain studies [10,16].

The presence of extracapsular extension (ECE) in tumors has consistently been identified as a negative prognostic factor. Its occurrence has been associated with poorer outcomes and an increased likelihood of disease progression. Therefore, monitoring for the presence of ECE is crucial when assessing the prognosis of individuals with head and neck cancer [10,16].

Smoking and excessive alcohol consumption are well-known causal factors for head and neck cancer. These behaviors have been firmly established as major risk factors for the development of this type of cancer. Consequently, addressing and mitigating these risk factors through targeted interventions, such as smoking cessation programs and alcohol abuse prevention measures, are essential in reducing the incidence and impact of head and neck cancer in affected populations [1,10,16].

Limitations

This systematic review has some limitations, such as its retrospective nature, the limited use of HPV testing in some studies, the inherent variability in staging workup in any multiple center series, and the inherent challenges of retrospective registry recorded data with a diagnosis of unknown primary of any disease site. Analyses that include information on local and regional control may be more valuable in determining the best treatment option. However, using these data sets makes it possible to analyze a larger population of this unusual entity than is possible with any single institution series. Another limitation of this study was that it only considered articles published in the last 10 years. Furthermore, as there was not an overwhelming amount of data on this topic, more in-depth research is necessary to draw more specific conclusions. This review may have missed articles in languages other than English and Spanish; however, despite a thorough search and usage of databases in Spanish, zero articles were found that were written in Latin America and the Caribbean, which further evidences the depth of the gaps in research on head and neck cancer in this population. Future studies should be conducted tailored to this community so that comparisons can be made and advancements can be implemented.

Evaluation of Bias of Selected Studies

Due to the heterogeneity in measurements and methodology, especially in terms of therapeutic approaches that could impact overall survival and outcomes, or the lack of inclusion of smoking status, the studies used for the final synthesis may have had information biases that could have led to an overestimation of the effect of HPV status and prevalence on patient outcomes in HNCUP. Results with a magnitude bigger than the actual one might emerge from these studies' common lack of blinding for the assessor and the patients, as well as the inter-study heterogeneity in outcome measurement. However, in other more controlled circumstances, there would likely be a probable reduction in the apparent effect while keeping a clinically meaningful influence. The overall quality of the included research was evaluated and was determined to be moderate in all articles, using the Newcastle-Ottawa scale for quality assessment of nonrandomized studies [33], rendering them idoneous for analysis.

## Conclusions

In conclusion, this systematic review provides important insights into the role of HPV in HNCUP. The review reveals that HPV is present in a significant proportion of HNCUP cases and highlights the relevance of considering HPV in diagnostic and treatment strategies, such as locating the primary tumor site. The findings also suggest a potential association between HPV and improved clinical outcomes in some studies, although the evidence is not consistent.

Additionally, given the increasing incidence of HPV-related HNCUP, further research is necessary to better understand the impact of HPV on this disease. Exploring the potential implications of HPV prevention, especially HPV vaccination and its effects on HNCUP is an important area for future investigation. Understanding the long-term effectiveness of HPV vaccination and its potential role in reducing HPV-related cases of HNCUP can contribute to preventive measures and public health strategies.

The importance of conducting additional research is crucial to enhance our knowledge about the role of HPV in HNCUP, particularly in diverse contexts, including developing countries. Such research efforts can lead to the development of innovative diagnostic approaches and treatment strategies that have the potential to improve the prognosis and outcomes of HNCUP patients. Therefore, ongoing research endeavors are essential to advance our understanding and refine our approaches to diagnosing and treating HNCUP.
